# Potential Role of Occupational Therapist Intervention in Elementary School for Children with Additional Support Needs: A Systematic Review

**DOI:** 10.3390/children10081291

**Published:** 2023-07-27

**Authors:** Marta Elisa Seoane-Martín, María Carmen Rodríguez-Martínez

**Affiliations:** 1 Hospital Psiquiátrico Penitenciario de Sevilla, 41006 Sevilla, Spain; 2 Department of Physiotherapy, Faculty of Health Sciences, University of Malaga, 29071 Malaga, Spain; 3 Instituto de Investigación Biomédica de Málaga y Plataforma en Nanomedicina—IBIMA Plataforma BIONAND (IBIMA Plataforma BIONAND), 29590 Malaga, Spain

**Keywords:** occupational therapy, primary school, learning disabilities

## Abstract

(1) Background: The main activity children engage with is learning through play or formal education. The aim of this systematic review is to analyze the role of occupational therapy in the school setting for children with additional support needs or disabilities. (2) Method: We conducted a systematic review using the preferred reporting items for systematic reviews and meta-analyses (PRISMA) guidelines. This systematic review was registered in PROSPERO (CDR42022314271). The search was performed in the following databases: ERIC, Dialnet Plus, PubMed, OTseeker, Cochrane, Scopus, CINAHL, and PsycINFO. (3) Results: In total, 1954 studies were identified, from which 18 articles were selected. These studies were heterogeneous and showed different types of intervention of the occupational therapist in school environments. (4) Conclusions: The main conclusions highlighted the effectiveness of the occupational therapist within the school environment, the importance of an interdisciplinary team to cover the special needs students within the school and the need for intrinsic motivation for an active and inclusive participation of the students with special needs. However, there is a need for more homogeneous studies with a larger sample size that specifically focus on the school context and include the involvement of occupational therapists in order to replicate the findings obtained.

## 1. Introduction

The primary objectives of occupational therapy are to enhance individuals’ capacity to engage in desired, necessary, or prescribed occupations across their lifespan [1]. Occupational therapists use the knowledge related to the person’s interaction, engagement with occupations, and context, designing an intervention that facilitates change or growth in factors such as body functions, values, beliefs, spirituality, and skills (motor, processing, and social interactions) to achieve successful user participation [2]. In this way, occupational therapy ensures that there is a balance between the person and his or her daily activities through the use of their occupation. In the case of children, their main activity is to learn either through play or through formal education. Taking this into consideration, the occupational therapist can intervene in the school context. Some of the purposes of occupational therapists in the school environment include helping students reach the desired academic outcome or compensate for learning difficulties with the aim of achieving their maximum potential [3]. The difficulties presented by children can originate from diverse dysfunctions, which can include motor impairments, a lack of verbal skills, and sensory alterations [4].

In Europe, specifically Switzerland, the role of occupational therapists is included as a part of the school system even though it can originate from outside the school; however, there exists permanent communication with educators [5]. In South America, the legislation applied in Argentina should be highlighted. Law 26.378 of the Convention on the Rights of Persons with Disabilities and its optional protocol, specifically article 24 on Education, underlines the importance of teachers trained in other methods of communication (braille, alternative and augmentative communication) along with other professionals working at different educational levels [6]. Additionally, the Chilean legislation established by the Ministry of Education on the Technical Cabinet of Special Schools detailed in Decree No. 363/1994 includes, in article four, the occupational therapist as a professional within the technical cabinets to support special education students, emphasizing the term multi-professional team [7]. After this international analysis of the situation of occupational therapy in schools, the purpose of this research is as stated. The aim of this review is to understand the effectiveness of occupational therapy in the school setting and its impact on the education of children with special needs.

At present, Spanish education is regulated by the Organic Law 3/2020 of 29 December on Education (LOE) [8] and the Organic Law 8/2013 of 9 December 2013 for the improvement of educational quality (LOMCE) [9] even though this one has been repealed and joined into the first one. These regulations include the general principles of education, including educational inclusion. Following this regulation, Spanish education is governed by the principles of standardization and inclusion, ensuring nondiscrimination and equality as regards access to and permanence in the educational system for the schooling of students with special needs [8]. More specifically, in the Autonomous Community of Andalusia, together with the LOMCE, the importance of Law 17/2007 of 10 December, on Education in Andalusia (LEA) establishes, in Title II on equity in education, that attention should be devoted to students with SEN (specific educational support needs) and to determine who these students are [10].

Nonetheless, both in the Spanish legislation and the regional legislation of Andalusia, there is no law that covers the role of the occupational therapist. There are some professional associations in other regions, such as Castilla-La Mancha, Navarra, Galicia, the Balearic Islands, Aragón, and La Rioja that have prepared documents justifying the role of the occupational therapist. The Basque Country community stands out as a pioneer in introducing occupational therapists among the support staff for students with special educational needs [11]. According to the World Federation of Occupational Therapists (WFOT), the United States was among the first countries to form this professional union [12]. They not only pioneered the introduction of occupational therapy in the country but also created legislation that protects them, as shown by the U.S. Department of Education’s The Education for All Handicapped Children Act 1975 (EAHCA or EHA) statute [13]. Chapter 33 on the education of persons with disabilities defines “related services” as transportation, developmental services, corrective and other support services, including occupational therapy, which are provided in order to ensure a free and appropriate public education for children with disabilities [14].

## 2. Materials and Methods

### 2.1. Search Strategies

A systemic review was carried out following the guidelines of preferred reporting items for systematic reviews and meta-analyses (PRISMA), considering the studies published between 2015 and 2022 in the following databases: ERIC, Dialnet, PubMed, OTseeker, Cochrane, Scopus, CINAHL and APA PsycINFO. The search was carried out using the following keywords based on the health sciences descriptors (MESH/DECS): “occupational therapy”, “primary school”, “school”, and “learning disabilities”. This systematic review was registered in PROSPERO (CRD42022314271).

### 2.2. Eligibility Criteria

The inclusion criteria were as follows: Studies published between 1 January 2015 and 7 November 2022; participants included children between the ages of 6 and 12; atopic about learning disorders or problems at school; studies that included the role of the occupational therapist in the school (primary); and the language of the study was Spanish or English.

Exclusion criteria were as follows: review letters to the editor, conference abstracts, studies about special education in higher and lower primary education, and studies about interventions by other professionals that did not include the occupational therapist.

### 2.3. Study Selection

Two independent reviewers conducted a thorough assessment of studies to determine their suitability for inclusion based on the predetermined eligibility criteria.

### 2.4. Quality Assessment of Included Studies

Three scales were used to minimize the risk of bias according to the type of study analyzed. CONSORT guidelines 2010 [15] were used to assess the internal validity of the studies in this systematic review. This tool is made up of a 25-item checklist that provides the author with standards on the trial’s design, analysis, and interpretation of the results, and there is no consensus about the score obtained as it is used as a guide instead of a test. The second, the AMSTAR-2 scale [16], was applied to systematic reviews, analyzes 16 items in total (compared to 11 in the original), includes a more comprehensive user guide, and has an overall rating based on weaknesses in critical domains. Finally, for observational studies, we used the Strengthening the Reporting of Observational Studies in Epidemiology (STROBE) checklist [17]. The STROBE statement is a checklist consisting of 22 items considered essential for the proper communication of observational studies [18] and is considered acceptably reported when at least 50% of the answers are positive (after removing “not applicable” items).

## 3. Results

**Figure 1:**
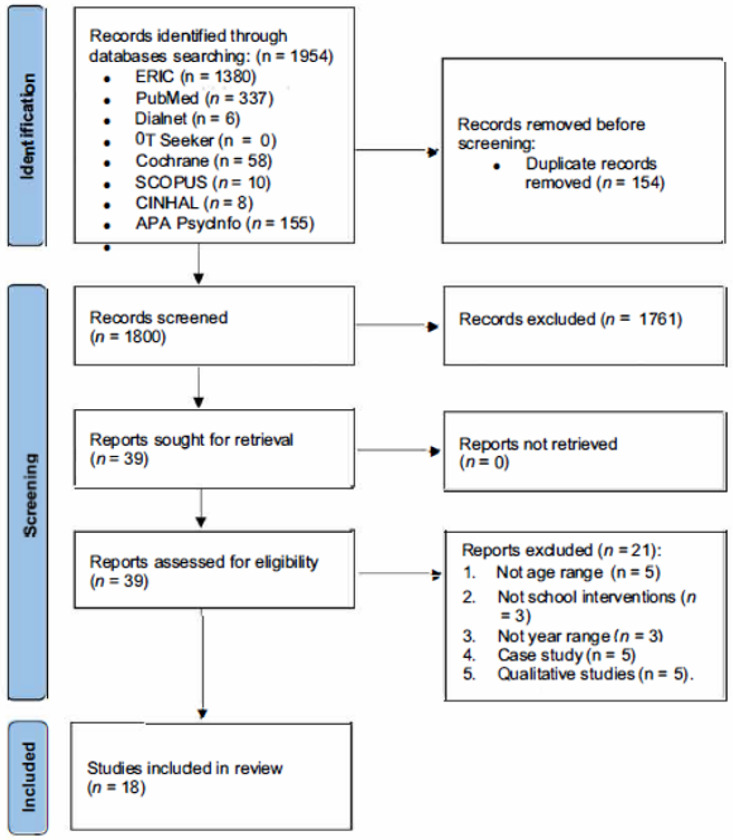
PRISMA flowchart of studies selection.

After removing duplicate records, 154 were reviewed screening titles and abstracts, but only 39 were selected. These studies were analyzed by title and abstract, of which 39 were selected by full text (Figure 1). Finally, after analysis of the remaining 39 studies, the total of selected articles was 18 because they met the inclusion criteria and were included in this review. The types of studies were three systematic reviews, three literature reviews, five pilot studies, five experimental studies (ECAs and pre/post-test), and 2 case–controls. The most relevant information from the articles obtained is summarized in Table 1 and includes the author, type of study, objective, sample, methodology, results and conclusions, and quality of the study.

### 3.1. Systematic Reviews

Three systematic reviews were identified. Firstly, Stauter et al. [19] employed The Evaluation Guidelines for Rating the Quality of an Intervention Study [20] and the Oxford levels of evidence [21] for their analysis. Secondly, Bray et al. [22] conducted their analysis using Downs et al. [23] and Ebell et al. [24]. Lastly, Fancher et al. [25] followed the PRISMA guidelines [26] and incorporated the American Academy for Cerebral Palsy and Developmental Medicine (AACPDM) in their study [27].

### 3.2. Narrative Reviews

Outside the framework of systematic analysis, we encountered narrative reviews that highlight the significance of education following the Montessori model, with Luborsky [28] emphasizing the benefits and advocating for occupational therapists as professionals who excel in meeting the diverse needs of students in the classroom. Based on the same philosophy, Strong et al. [29] analyzed the importance of an approach that does not focus exclusively on formal education but includes other elements, such as motivation or social participation. However, the results found were not significant. Additionally, Anaby et al. [30] conducted the search strategy proposed by Arksey et al. [31], concluding that collaborative services could provide guidance to rehabilitation practitioners in enhancing care promotion, thus facilitating participation and inclusion in school settings.

### 3.3. Pilot Studies

Concerning pilot studies, Grajo et al. [32] first proposed an intervention centered around reading as a meaningful occupation for children. The analysis also incorporated the following standardized tests: Gray Oral Reading Tests, fifth edition [33]; Test of Word Reading Efficiency, second edition [34]; Woodcock Reading Mastery Test, third edition [35], as well as qualitative evaluations; Canadian Occupational Performance Measure fourth edition [36] and Inventory of Reading Occupations [37]. Undoubtedly, the use of media in learning is highly increased. Studies not only aim to transform the dynamics of learning in schools but also to integrate technological resources that facilitate learning, particularly for students with special needs. Despite the limited sample size, Hettiarachchi et al. [38] implemented multisensory storytelling activities, observing a positive trend in expressive vocabulary skills.

Secondly, Siyam et al. [39] employed a two-part approach, starting with a participatory design methodology to identify fundamental design principles. These principles were subsequently utilized in designing and implementing a mobile app called IEP-Connect. Lastbut certainly not least, Menin et al. [40] proposed the utilization of e-books as a medium for content transfer in special education classrooms, despite the limited sample size.

### 3.4. Experimental Studies

In this systematic review, we also included randomized controlled trials, such as Esmaili et al. [41] and a protocol study by Romero-Ayuso et al. [42]. These studies shared some characteristics; they were single-blinded, and participants were randomly assigned to the intervention and control groups using random-number tables. However, the second one used an online program. Continuing with the quasi-experimental studies, Patton et al. [43] and Patton et al. [44] conducted pre- and post-intervention assessments, advocating for the use of *Handwriting Without Tears* in children with Down Syndrome [45] and emphasizing the importance of occupational therapist involvement, as also promoted by Lee et al. [46]. Another study examined a method for enhancing handwriting skills by utilizing the Size Matters Handwriting Program, resulting in improvements across a minimum of two quality categories of handwriting [47].

Another practical approach was *Handwriting Without Tears* [45] through the utilization of *Keyboarding Without Tears*^®^ *(KWT)* [48]. Donica et al. [49] proposed that this alternative method, advocated by occupational therapists, serves as an augmentative or alternative means of learning, particularly beneficial for children with special needs.

### 3.5. Observational Studies

According to case–control studies, as was previously mentioned, a sensory-based approach is currently being researched as complementary to traditional methods. Wild et al. [50] proposed a collaborative intervention with the occupational therapist addressing sensory disorders at school. On the other hand, Şahin et al. [51] chooses a general approach to studying the Participation Environment Measurement for Children and Youth (PEM-CY). It analyzed environmental factors at home, school, and community levels, comparing children with learning disabilities and those who are non-disabled.

Table 1 shows the studies with occupational therapist interventions at school. All the characteristics of the studies that have been included are specified below.

The findings derived from the analysis of the research articles indicated a generally limited quality rating, primarily attributed to small sample sizes and the variability of the occupational therapist’s role within the school setting. Nonetheless, the research proved to be informative and valuable for practitioners, given the extensive range of intervention possibilities and diverse practice areas. Evidently, all children with learning difficulties benefited from the occupational therapist’s interventions within the school or, alternatively, as part of multidisciplinary teams. The synthesis of the studies revealed commonalities in successful educational strategies, participation outcomes, and emerging literacy achievements.

**Table 1:** Description of Studies.

Author and Year	Type of Study	Objective	Population/Sample	Methodology	Results and Conclusions	Quality
Stauter, Myers & Classen, 2017 [19]	Systematic review	Offer teaching strategies that include the use of augmentative and alternative communication (ACC) models, adaptive materials, subvocal word/phoneme testing, contextual learning, and differentiated teaching.	286 items	The search for articles in the years 2005–2015. The following databases were searched: Science Direct, Sage Premier, PsycINFO, PubMed, PsycARTICLES, MEDLINE Complete, JSTOR, HaPI, ERIC, Education Research Complete, Dynamed, Cochrane, CINAHL, and Academic Search Premier. A total of 122 outcomes were identified.	The use of these strategies improves the development of literacy in children with physical and speech disabilities (SSPI). All professionals must assume that these children are capable of developing these skills, improving social participation.	AMSTAR-2 scale: 6/16
Bray et al., 2021 [22]	Systematic review	Focusing on handwriting and/or spelling interventions for children with SLD	365 items for handwriting, 251 items for spelling	The search for articles in the years 2008–2020, The following databases were searched: Academic Search Complete, CINAHL, ERIC, MEDLINE, Psychology and Behavioural Sciences Collection, APA PsycINFO, and the Teacher Reference Center.	The evidence gathered in this systematic review provides moderate overall support for the effectiveness of existing interventions targeting handwriting and/or spelling skills in children with specific learning disabilities (SLD). Particularly notable clinical outcomes were observed with the occupation-as-means approach, specifically emphasizing self-reflection and self-correction techniques.	AMSTAR-2 scale: 9/16
Fancher et al., 2018 [25]	Systematic review	The influence of early writing skills in preschool-aged children and the efficacy of handwriting interventions from kindergarten to second grade.	256 items	The search for articles in the years 2000–2017. The following databases were searched: Cochrane Database of Systematic Reviews, ERIC, PubMed, CINAHL, Academic Search Elite, and Ovid databases.	Support the need to retain handwriting instruction in primary grades. The strength of the available evidence provided the basis for handwriting to be an evidence-supported practice and potentially a universal design that can be provided in classroom contexts.	AMSTAR-2 scale: 6/16
Luborsky, 2017 [28]	Literature review	This article examines the involvement of occupational therapists in assisting Montessori teachers in comprehending and addressing the requirements of students experiencing attention challenges within their classrooms.	Children with attention problems	Not applicable	The individualized program may include changes in the environment, teaching, materials, and/or the addition of supplementary equipment and materials. One of the most relevant professionals is the occupational therapist, whose purpose is to attend to the needs of these students in the classroom.	Not applicable
Strong et al., 2018 [29]	Literature review	To emphasize the significance of research and available materials that occupational therapists can utilize in their daily practice to support and enhance the literacy skills of the children they serve.	Not applicable	Not applicable	First, sensory and visual-motor needs are considered. Second, support is provided for reading (motivational books), writing (songs and rhymes), and listening (sensory diet).	Not applicable
Anaby et al., 2018 [30]	Literature review	(1) Synthesize current evidence about principles for organizing and delivering interdisciplinary school-based support services for students with disabilities and (2) Ascertain helpful strategies for implementation of principles in the school setting.	531 items	The search for articles in the years 1998–2017. The following databases were searched: Medline, CINAHL, PsycINFO, ERIC, and ProQuest.	The findings have the potential to inform rehabilitation professionals, educators, and policymakers in establishing coordinated and collaborative services that prioritize training and capacity-building for school-based service providers.	Not applicable
Grajo& Candler, 2016 [32]	Pilot clinical application case study	The purpose of the OPARI is to supplement skill-based reading interventions. Improving participation and engagement in reading as an occupation.	From a pool of 15 children from two private elementary schools in St. Louis, MO, who were identified from their participation in a previous study concerning the phenomenological experiences of struggling readers, five children were invited to participate based on the inclusion criteria	The five children completed the initial evaluation two weeks prior to the start of the therapy program. Parents were invited to participate during the initial evaluation. The therapy program consisted of 7 weeks of pull-out therapy sessions, two sessions per week for a total of fourteen sessions. Each week, the children participated in a group session with all participants for 90 min and a 60 min individualized therapy session.	The reading-abilities scores indicate no statistical difference during the 8-week therapy program. However, statistically and clinically significant increases (an average of 3 COPM points for performance and 5 COPM points for satisfaction) were noted in the individual children’s perception of their performance and satisfaction with performance with their self-chosen reading goals.	Not applicable
Hettiarachchi et al., 2020 [38]	Pilot study	To promote the use of multisensory stimuli to advance the development of communication and language skills in children with intellectual disabilities.	7 children	Vocabulary measures of word naming of target vocabulary were undertaken pre- and post-intervention using picture-based tasks presented via PowerPoint.	The results indicate the potential advantages of employing culturally relevant and familiar local traditional narratives, accompanied by a diverse array of multisensory stimuli and storytelling activities, to facilitate the acquisition of new vocabulary among children with disabilities who are learning English as an additional language.	Not applicable
Siyam & Abdallah, 2021 [39]	Pilot study	To examine the utilization of mobile technology as a means of coordinating therapy and facilitating learning for students with special education needs and disabilities (SEND).	120 students with SEND	The participatory design research methodology follows an iteration of three main stages; initial exploration of work, discovery process, and prototyping.	The findings from the usability evaluations demonstrated that the application exhibited a high level of usability and user satisfaction. Additionally, the app was perceived as efficient, effective, user-friendly, and beneficial.	Not applicable
Menin, Perham, Vong & Wachtel, 2016 [40]	Pilot study	To benefit students with difficulties in the creation of e-books. Another objective is to increase the link between the subject matter of general classes and specific classrooms.	4 students	Students created the storyboards for 3 weeks and then scanned them using PowerPoint. During the following sessions, they added text and images and, finally, the recording of the narration.	E-books provide an opportunity for students receiving special education to participate in age-appropriate activities and share educational experiences with general education peers. Occupational therapists can promote the creation of e-books in general education classrooms.	Not applicable
Esmaili et al., 2019 [41]	Randomized controlled trial	The aim of this study is to utilize the Model of Human Occupation to explore the impact of peer-play activities on occupational values, competence, and executive function skills (specifically, behavior regulation and meta-cognition) among children diagnosed with specific learning disabilities (SLD).	49 children	Students diagnosed with specific learning disabilities (SLD) were assigned randomly to either the peer-play group or the control group. The assessment tools employed in this study were the Behavior Rating Inventory of Executive Function and the Child Occupational Self-Assessment (COSA).	Data analysis showed that the effects of the intervention on EF skills were medium to large. The occupational values and competence did not change according to the COSA.	CONSORT reporting guidelines: 18/25
Romero-Ayuso et al., 2020 [42]	Protocol study	To improve self-regulation skills in children between 6 and 11 years of age with neurodevelopment disorders.	Not applicable	A randomized controlled trial will be conducted with the use of “SR-Mrehab. An assessment will be conducted before and after the intervention and 24 weeks after the end of the intervention process. The experimental group will receive the intervention using virtual reality. The control group will receive a standard self-regulation program.	Changes in self-regulation, as well as the acceptability of technology with the use of SR-Mrehab, will be evaluated. The results will be published and will provide evidence regarding the use of this type of intervention in children with neurodevelopment disorders.	Not applicable
Patton & Hutton, 2016 [43]	Pre-test/post-test	To present pre- and post-intervention data relevant to the profile of writing readiness in children with Down Syndrome (DS) from a larger doctoral study.	28 children	Data were collected in 2006–2007. Forty-six children with DS attending mainstream schools in 3 counties in the Republic of Ireland and their parents and teachers were recruited using purposive sampling.	Teacher and parent reports highlighted the need for collaborative intervention with occupational therapy. Findings from the study support the need for targeted early collaborative syndrome-specific intervention to support the development of writing readiness in children with DS as an important part of school readiness.	CONSORT reporting guidelines: 14/25
Patton & Hutton, 2017 [44]	Pre-test/post-test	To provide an evaluation of the HWT (Handwriting Without Tears) method applied as an intervention to promote handwriting among children with Down Syndrome attending mainstream school in the Republic of Ireland.	40 children	Mixed methods were used in this descriptive evaluation of the application of the HWT method with children with Down Syndrome. The HWT program was delivered over a period of 8 months, from October 2006 to June 2007. Data were gathered from a structured observation of group HWT sessions with the children using a purpose-designed HWT task-participation scale.	Positive changes in participation in HWT activities were recorded in group data and in teacher/parent reports. Tentative findings suggest that hands-on multisensory learning approaches such as HWT may encourage children with Down Syndrome to participate in activities that promote handwriting skills.	CONSORT reporting guidelines: 10/25
Lee & Lape, 2019 [46]	Pre-test/post-test	To determine whether a cognitive approach to handwriting combined with multiple self-monitoring strategies, embedded into a second-grade classroom curriculum with teacher and occupational therapist collaboration, was effective for improving handwriting legibility and students’ perceptions of self-monitoring strategies.	19 students	Students’ handwriting legibility was assessed using the Minnesota Handwriting Assessment prior to and immediately following the 6-week implementation period of the Size Matters Handwriting Program. Students also completed a self-assessment of their perceptions of self-monitoring skills after the implementation period.	Students’ perceptions of self-monitoring, collected via a brief self-report measure, reveal that most students agreed that self -monitoring played an essential role in their learning by understanding the rules of handwriting and learning proper spacing strategies when writing. These results support the effectiveness of employing the Size Matters Handwriting Program and teaching self-monitoring strategies.	CONSORT reporting guidelines: 9/25
Donica, Giroux & Faust, 2018 [49]	Pre-test/post-test	To examine the effectiveness of a developmentally based curriculum, Keyboarding Without Tears^®^, as compared to free web-based activities for learning keyboarding skills in students.	Total of 1908 students, data from 4 elementary schools, from kindergarten through fifth	KWT offers a grade-based 36-week curriculum designed for instruction in 5–10 min a day or 30 min a week, targeted for grades kindergarten through fifth. Free Web-Based Activity Instruction: the lessons included 30 different two-key combinations for rote practice, while the games offered 40 combinations of letters.	Inter-rater reliabilities for keyboarding method observation among three (pre-test) and four (post-test) raters were excellent (ICC = 0.97 and 0.98, respectively), suggesting the keyboarding method observations were scored similarly among the researchers.	CONSORT reporting guidelines: 14/25
Wild &Steeley, 2018 [50]	Case-Control study	To examine the efficacy of a classroom-based program (henceforth referred to as the“BrainWorks Program”) for children with sensory processing challenges.	261 students	Students were divided by age and degree of sensory needs between control and experimental groups, with teachers of students in the experimental group implementing the recommended sensory program (BrainWorks) with all students in the classroom.	Pre- and post-analysis of data based on both the SPM and the BASC-2 showed significant improvement. The control group, on the other hand, reflected no interventions beyond pre-existing work based on IEP goals to the extent that it was being implemented.	STROBE scale: 15/22
Şahin et al., 2020 [51]	Case–Control study	To examine the participation and environmental features of children with specific learning disabilities (SLD) compared to non-disabled children.	178 students	The Participation Environment Measurement for Children and Youth (PEM-CY) is a parent-reported instrument used to assess children’s participation as well as environmental factors in the home (watching TV/videos, personal care management, homework, Etc.), at school (classroom activities, field trips, and school events, getting together with peers outside of class, Etc.), and in the community (neighborhood outings, organized or unstructured physical activities).	This study provides information about participation patterns and environmental factors for many children with SLD. The results provide insights into a rehabilitation program that may improve the participation of children with SLD and where more outstanding greater efforts are needed to support participation and environmental features for children with SLD.	STROBE scale: 15/22

## 4. Discussion

This systematic review aimed to analyze the current status of occupational therapists’ role in the school environment and its impact on the education of children with disabilities. The 18 articles selected were subsequently analyzed and categorized based on their respective subject areas, according to our objectives. The obtained results were not homogeneous, possibly attributed to the diverse range of areas within schools where occupational therapists can provide interventions.

### 4.1. Participation

Stauter et al. [19] focus on the use of augmentative and alternative communication (AAC) models, and their findings align with the evidence of Strong et al. [29] regarding the impact of the intervention on social participation. These studies analyze literacy, and although the results are not statistically significant [19,29], both emphasize the importance of an approach that goes beyond formal education and incorporates elements such as motivation or social participation [19,29]. It is also confirmed that peer-play activities have a positive effect on executive function, especially for children with specific learning disabilities [41].

Authors should discuss the results and how they can be interpreted from the perspective of previous studies and the working hypotheses. The findings and their implications should be discussed in the broadest context possible. Future research directions may also be stressed.

Inclusive educational environments are one of the top priorities in every educational system, but, indeed, it is not a real functional option yet. It was analyzed by Anaby et al. [30] that various professionals are involved in providing services in the majority of cases related to behavioral concerns (23%), followed by learning disabilities (12%). One common aspect of all the mentioned studies is the coeducational strategy. Furthermore, the need to include other professionals, such as the occupational therapist’s role, is also highlighted so that they work together with the teachers for the child’s optimal development [30].

### 4.2. Literacy Skills

The utilization of conventional learning methods can present challenges for children across the board, and adopting alternative approaches to learning has the potential to enhance their performance [46]. There are many types of interventions related to handwriting, typing, visual–perceptual, and sensory-motor activities, among others [25]. One common intervention applied in SLD, specifically in Down Syndrome, is *Handwriting Without Tears* [43,44], which distinguishes the importance of having an occupational therapist in the intervention due to the scarce training of teachers in handwriting to children with SLD. It is shared and complements the vision of an unconventional approach, more individualized intervention, and includes the occupational therapist as part of the professional team in charge of using occupational participation and commitment to strengthen children’s ability to make them succeed in reading [22,32].

Another alternative is *Keyboarding Without Tears*, developed from the original intervention *Handwriting Without Tears*, which can be used with general or special education students. It is also an adaptation for those with physical impairment [49]. Menin et al. [40] indicated that the occupational therapist is a necessary figure in schools to support special education students, and hence the proposal to create e-books, not only to facilitate equal opportunities for these students but also to bring them closer to the rest of their peers by sharing the educational experience. Another proposal could be a multisensory traditional storytelling program whose main objective is to improve vocabulary development in SLD [38].

The contribution of occupational therapy to literacy is highly extensive, as it can intervene when there are organic or neurological challenges in the processing or execution of the activity [2].

### 4.3. Educational Models

Beyond the confines of traditional and antiquated educational structures, Luborsky [28] underscores the advantages of adopting an educational framework based on the Montessori model. In this regard, Luborsky advocates for the inclusion of occupational therapists as highly qualified professionals capable of addressing the diverse needs of students in the classroom. Another unconventional approach is the sensory model, wherein not only Sensory Integration is gaining popularity but also the associated concepts it encompasses. One such concept is self-regulation, which pertains to the capacity to manage and regulate behavior, including emotional and cognitive aspects. This ability holds significant relevance in neurodevelopmental disorders [42].

Finally, Virtual reality could promote engagement in intervention easily and, at the same time, changes in self-regulation in children with special educational needs, as indicated by recent studies [42].

It is necessary to emphasize that the occupational therapist holds the exclusive professional qualification to address issues about sensory integration. Consequently, they are the only professional entitled to establish an intervention in any environment, including the school [2].

## 5. Conclusions

One of the most relevant conclusions of this systematic review is the effectiveness of occupational therapists within the school setting. Primary objective achievements are the significant findings from all the analyzed studies that were considered, encompassing an evaluation of the favorable and unfavorable aspects of the therapist’s engagement in schools and their interaction with children with disabilities. The following conclusions can be drawn following the framework structure employed in the discussion. 

### 5.1. Participation

Although the selected studies had a reduced sample, most of them highlighted the occupational therapist’s relevance and the importance of an interdisciplinary team to cover the special needs within the school. It includes the intervention within the environment, in this case, the classrooms with the children. Additionally, it is necessary to provide a degree of intrinsic motivation for the active and inclusive participation of children with special needs. It should also be noted that the therapist could intervene with students without special needs, but they need to organize routines, habits, etc., to obtain a generally positive effect.

### 5.2. Literacy Skills

The literature reveals the transformations in the educational approaches for children, particularly those with learning difficulties. Although the advantages of alternative teaching methods, such as those employed in literacy, are widely acknowledged, their widespread implementation may not be readily apparent. Nevertheless, the emergence of novel techniques and instructional approaches has expanded the scope of professionals involved in education beyond traditional teachers, notably occupational therapists.

### 5.3. Educational Models

In general, one of the aspects that must be highlighted is the effect of the intervention on neurotypical children since some studies also analyzed this population. This data could lead to a reflection on the benefits of occupational therapy applied to all students, both those with unique needs and neurotypical students. Specifically, researchers have highlighted the deficits observed in typical developing children and the potential advantages of emerging technologies, which present therapeutic applications in the school setting. Consequently, studies have demonstrated the benefits of occupational therapy for children with learning difficulties, aiming to assess its effectiveness in improving outcomes for both groups of children.

### 5.4. Limitations

Finally, several limitations have been identified in this review. One limitation is the need for more homogeneity in the literature on the subject. Additionally, other limitations were observed regarding the scope of occupational therapy within the classroom and the types of studies that consider the occupational therapist’s role. These limitations may stem from the versatile nature of the occupational therapist’s role. It is essential to emphasize the lack of evidence specifically within Spain, primarily due to the absence of nationwide recognition of occupational therapists in primary schools according to Spanish legislation. However, some professional associations are actively working to address this issue and achieve recognition for occupational therapists comparable to that in the public health system.

## Data Availability

Data are available upon request to the authors.
